# Pediatric Neurocysticercosis: Usefulness of Antibody Response in Cysticidal Treatment Follow-Up

**DOI:** 10.1155/2014/904046

**Published:** 2014-08-06

**Authors:** Venkata Subba Rao Atluri, Venkateswara Reddy Gogulamudi, Pratibha Singhi, Niranjan Khandelwal, Lakshmana Swamy Parasa, Nancy Malla

**Affiliations:** ^1^Department of Immunology, Herbert Wertheim College of Medicine, Florida International University, Miami, FL 33199, USA; ^2^Department of Parasitology, Postgraduate Institute of Medical Education and Research, Chandigarh 160012, India; ^3^Department of Paediatrics, Postgraduate Institute of Medical Education and Research, Chandigarh 160012, India; ^4^Department of Radiodiagnosis and Imaging, Postgraduate Institute of Medical Education and Research, Chandigarh 160012, India; ^5^Department of Veterinary Public Health, NTR College of Veterinary Science, Gannavaram, Andhra Pradesh 521102, India

## Abstract

Serum and urine samples were collected from 33 NCC patients before the albendazole treatment, 3–6 and 12 months PT. At 3 months PT, 24 (72.7%) patients had no detectable CT/MRI lesions and 9 (27.2%) patients had persistent lesions. Antibody response to crude soluble extract (CSE), excretory secretory (ES), and lower molecular mass (LMM) (10–30 KDa) antigenic fraction of *T. solium* cysticerci was detected in serum and urine samples by ELISA. Before the treatment, out of 33 NCC children, 14 (42.4%), 22 (66.6%), and 11 (33.3%) serum samples were found positive with the use of CSE, ES, and LMM antigen, respectively. At 3–6 months PT, positivity rate was 5 (15.1%), 2 (6%), and 4 (12.1%) and at 12 months PT, positivity rate was 5 (15.1%), 0, and 3 (9%) with the use of CSE, ES, and LMM antigen, respectively. There was no significant difference in the positivity with the use of three antigens in pretreatment and PT urine samples. The study suggests that the use of ES antigen to detect antibody in serum samples may serve better purpose to evaluate the therapeutic response in patients with NCC.

## 1. Introduction

Neurocysticercosis (NCC) is caused by the presence of* Taenia solium* larvae, the cysticerci in central nervous system, and is the most common cause of epilepsy in India. It is estimated that* T. solium* cysticercosis affects 50 million people worldwide [1]. Conservative estimates describe 50,000 deaths every year due to neurocysticercosis [2]. It is regarded as a major public health threat and economic burden in the developing countries of Asia, Africa, and Latin America. Important foci exist in USSR, India, China, Pakistan, Philippines, and Indonesia [3, 4]. Highest prevalence rates exist in communities where there is close contact between man and pigs, hygienic standards are low, and pork is eaten raw or undercooked. In a survey over a period of 20 years in north India, it was found in 5.9% of 103 epileptics and 11.1% amongst patients attending a neurology clinic in Postgraduate Institute of Medical Education and Research, Sawhney et al. [5]. The disease was earlier thought to occur less frequently in children and most of the available literature on NCC is concerned with adult patients [6]. Now the disease is being increasingly recognized in children [7]. Up to 50% of children with partial seizures in the Postgraduate Institute of Medical Education and Research (PGIMER) emergency service of the Nehru hospital, Chandigarh, India, have a recognizable underlying cause, the most common being neurocysticercosis and tuberculoma, both of which are amenable to specific pharmacotherapy [8].

Therapeutic measures for NCC can be considered as symptomatic treatment and definitive medical or surgical treatment. In the definitive therapy, for cyst destruction, antihelminthic drug albendazole has been used in a dose of 15 mg/kg/day in two or three divided doses for 28 days and shorter durations of 14 days to 8 days have also been used [9]. In a placebo-controlled trial of 1 week versus 4 weeks of albendazole therapy in children with one to three enhancing lesions, both regimens were found to be equally effective [10]. Resolution of lesion on CT scans at 3 months was seen in 68.3% and 68.8% in the 1-week and 4-week treatment groups, respectively. Seizure control at 1 year was similar in both groups. Although resolving of the active lesions on computer tomography was observed after 3 months of the treatment, cured patients remain seropositive even after one year of the treatment [11]. It indicates that persistent seropositivity does not necessarily indicate active infection. Although studies to evaluate the assessment of therapeutic response by detecting humoral immune response [12–14] and detection of antigens [15–18] in ELISA are available, serological methods to assess the therapeutic response in correlation with the radioimaging methods are scanty. Recently, HP10 antigen detection enzyme-linked immunosorbent assay was compared with the magnetic resonance imaging (MRI) in NCC follow-up patients [19]. Moreover, nonavailability of radioimaging facilities in many centers of endemic countries indicates the need to develop a serological method to evaluate the therapeutic response in NCC patients. Excretory secretory (ES) antigens are a complex mixture of proteins since they are metabolic products of live metacestodes; thus antibodies against ES can be considered as an indication of the presence of live parasite [20, 21]. The ES antigens have been found to be a better serodiagnostic antigen than crude antigen in other parasitic diseases [22–25]. In our earlier studies, we reported that the ES antigens are highly sensitive in both ELISA and EITB assay for the detection of antibody in serum samples for the diagnosis of neurocysticercosis in children [26–28]. The aim of the present study was to evaluate the efficacy of excretory secretory antigens in comparison to crude and lower molecular mass antigenic fractions in ELISA to assess the therapeutic response to albendazole in children with neurocysticercosis.

## 2. Materials and Methods

### 2.1. Patients and Controls

A cohort study was carried out in a tertiary hospital setting at the Advanced Pediatrics Centre (APC), the Department of Pediatric Medicine attached to the Postgraduate Institute of Medical Education and Research, Chandigarh, India. In total, 36 clinically suspected and radiologically proven NCC children and 5 children who attended outpatient department with minor illness (control) were enrolled in this study after taking due consent from the parents or guardians. Children with enhancing lesions in brain MRI are called active lesion NCC cases. The samples collected from 5 control children were used to assess the cut-off ELISA absorbance (OD) values. Serum and urine samples were collected from NCC children and controls.

Follow-up: children with NCC were given albendazole orally in a dose of 15 mg/kg/day for 28 days in case of multiple lesions or 8 days in case of single lesion patients. All the NCC patients were followed for one year and two follow-up samples were collected; one between 3 and 6 months and second sample at 12 months after the treatment. Patients were examined again clinically (for symptoms) and radiologically (for persistence of the lesion) at 3 months after ending the treatment. CT/MRI of the NCC patients was available only 3 months after completion of the treatment and was not done 12 months following treatment. Radiological evaluation of the pre- and posttreatment NCC children was done by the radiologists in the Department of Radiodiagnosis and Imaging, Postgraduate Institute of Medical Education and Research, Chandigarh, India. The radiologists were not aware of the ELISA results. Patients with the sustained enhancing lesions are considered as nonresponsive and patients with calcified lesions are considered as responders.

### 2.2. Preparation of the Antigens

Cysts were obtained from naturally infected pork from the local slaughter house, Chandigarh, India, and confirmed microscopically as* T. solium* cysticerci.

#### 2.2.1. Crude Soluble Extract (CSE Antigen)

Crude soluble antigen was prepared from the cysts isolated as detailed out earlier [28, 29]. Briefly, cysts were washed for 4 hours in phosphate buffer saline (PBS) (pH 7.4) containing antibiotics and antifungal agents. Cysts were suspended in normal saline and homogenized at 4°C in a tissue grinder to form a pulp. The pulp was sonicated and centrifuged and the clear supernatant obtained was stored at −20°C in 1 mL aliquots till further use.

#### 2.2.2. Excretory-Secretory Antigen

*T. solium *cysticerci separated from pork muscle were subjected to in vitro cultivation as detailedout earlier [28, 30]. Briefly, intact larvae with smooth translucent bladder wall and containing fluid were washed extensively for 2-3 hours in PBS and distributed into tissue culture flasks containing the RPMI 1640 medium followed by incubation at 37°C in 5% CO_2_. The medium was discarded after 12 and 24 hrs and replenished each time with fresh medium. Cell-free medium was collected every 24 hrs thereafter for 1 week. The membrane components were removed from the collected medium by centrifugation at 10,000 rpm for 30 min and the antigen was concentrated by precipitation in the 90% saturated solution of ammonium sulfate. The precipitate was dissolved in PBS and subjected to extensive dialysis against PBS (pH 7.2), overnight at 4°C and stored at −20°C for further use.

#### 2.2.3. Lower Molecular Mass Antigen Fraction (10–30 KDA)

The LMM antigen fraction was essentially prepared using electroelution as detailed out earlier [28]. Briefly, CSE antigen of* Taenia solium* cysticerci was fractionated by SDS-PAGE using 4% stacking gel and 15% separating gel under reducing conditions. By comparing the standard protein molecular weight marker, the gel corresponding to 10–30 kDa portions was cut and the antigen was eluted from the gel by using electroeluter. The antigen was further purified by dialysis, overnight at 4°C against PBS.

All the three antigens were estimated for the protein concentration just before use [31].

### 2.3. ELISA

The ELISA was carried out according to the standard method with slight modifications [28]. The optimum dilutions of the antigens, serum, and conjugate were determined by checker board titration. In the final set-up of ELISA, by using the results of checker board titration, all the serum samples were used at 1 : 400, 1 : 800, and 1 : 1600 dilutions and urine samples as undiluted. The optimum concentrations of the antigens were found to be 2 *μ*g, 1 *μ*g, and 0.002 *μ*g per well and conjugate dilutions, 1 : 40,000, 1 : 40,000, and 1 : 30,000 for both serum and urine with the use of CSE, ES, and LMM antigens, respectively. Each well of the 96-well microtiter plate (Nunc Inter Med, Denmark) was coated with 100 *μ*L optimum dilutions of the antigen in carbonate bicarbonate buffer and incubated at 4°C overnight followed by washing 3 times with PBS containing 0.02% Tween-20 (PBST). The nonspecific sites were blocked with 2%BSA in PBST and plate was incubated at 37°C for 1 hr followed by washing for 3x with PBST. Three dilutions (1 : 400, 1 : 800, and 1 : 1600) of the test, positive and negative control sera prepared in 1% BSA in PBST, and neat urine samples were added (100 *μ*L) to each well. Three positive, 5 negative control sera and urine samples, and one blank were included in each plate. These plates were incubated at 37°C for 1 hr and again washed 3x with PBST followed by addition of 100 *μ*L/well of optimum dilution of anti-human IgG conjugated with horse radish peroxidase (Sigma Aldrich) in 1%BSA in PBST and incubated at 37°C for 1 hr. Following incubation, plates were washed 3x with PBST and* ortho*-phenylenediamine and H_2_O_2_ were subsequently added as substrate (100 *μ*L/well). The plates were incubated in darkness for 15–30 min and the reaction was stopped by adding 3 M H_2_SO_4_. The absorbance of the contents of each well was read at 492 nm in an A4 ELISA reader (Eurogenetics, Tessenderlo, Belgium).

The cut-off absorbance value (OD) in each plate for serum and neat urine was determined by the mean absorbance of the 5 negative control sera/urine samples plus 2 S.D. The test sera/urine giving absorbance that was equal to and/or above the cut-off OD was considered to be ELISA positive at that dilution. Each sample was tested in duplicate.

### 2.4. Statistical Analysis

Positivity rate was calculated at the cut-off dilution (1 : 400) of the sera (to get 95% confidence interval) and neat urine samples. Significance in the difference of ELISA positivity in the samples collected before and after treatment was analyzed by using the McNemar Test Exact. A *P* value of <0.05 was taken as indicative of a statistically significant difference.

## 3. Results

### 3.1. Demographic and Radiological Characteristics of NCC Cases

#### 3.1.1. Pretreatment Evaluation

The age of the enrolled children in this study was between 3 and 12 years. Brain scans of 36 NCC children by CT/MRI, revealed single cysticercus granuloma (SCG) in 31 (86.1%) and multiple lesion neurocysticercosis (MLNCC) in 5 (13.9%) patients. Out of 31 patients with SCG, 29 (93.5%) had active (enhancing lesion) and 2 (6.5%) had calcified lesion and, out of 5 MLNCC cases, 4 (80%) had active and 1 (20%) had calcified lesions. The 3 children with calcified lesions were excluded from this study (2 children with SCG + 1 patient with MLNCC). All of the 33 (100%) NCC children presented with seizures and of these focal seizures (88.9%) were more common compared to generalized seizures (11.1%). The second most common clinical presentation was headache (44.8%) followed by nausea and vomiting (38.4%).

#### 3.1.2. Posttreatment (PT) Evaluation (Radiological versus Clinical)

To assess response to the treatment, CT was repeated 3 months after completion of therapy. Out of 29 SCG patients, the PT analysis showed that single active cyst persisted in 7 (24.1%) patients. Out of 4 MLNCC patients, multiple active cysts persisted in 2 (50%) patients. Twenty-four (72.7%) patients had no CT/MRI detectable lesion at 3 months PT. Thus, on the basis of repeat CT/MRI evaluation, 24 (72.7%) cases were categorized as responders and 9 (27.2%) with persistent lesion as nonresponders.

Out of 24 responders, 1 (4.1%) patient had persistent symptoms (headache) and, out of 9 nonresponders, two patients (22.2%) (one with headache and one with seizures and vomiting) had persistent symptoms even 12 months after the treatment.

### 3.2. Antibody Detection

#### 3.2.1. Serum before Treatment

**Figure 1:**
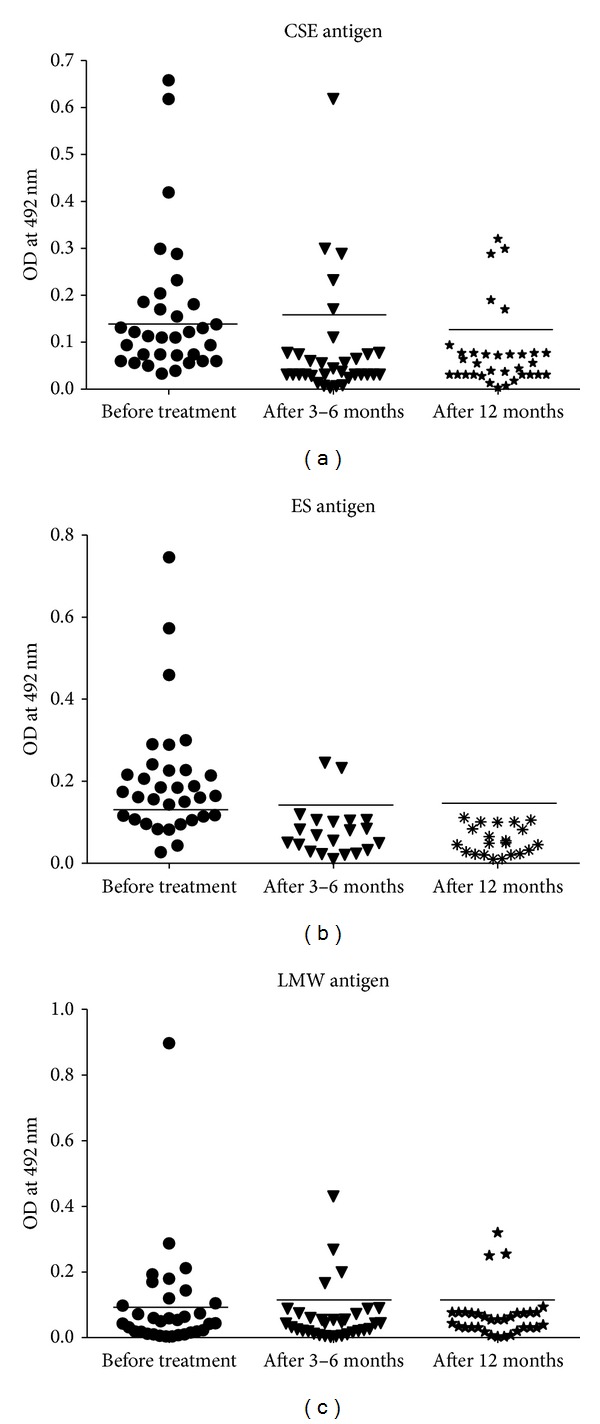
(a) Scattergram of absorbance measured by ELISA to detect serum antibody against CSE antigen in children with neurocysticercosis before and after the treatment: from 33 clinically suspected and radiologically proven NCC children, serum samples were collected before and after 3–6 months and 12 months completion of the treatment with albendazole. Antibody response against CSE was measured by using ELISA. Solid line indicates cut-off OD value. (b) Scattergram of absorbance measured by ELISA to detect serum antibody against ES antigen in children with neurocysticercosis before and after the treatment: from 33 clinically suspected and radiologically proven NCC children, serum samples were collected before and after 3–6 months and 12 months completion of the treatment with albendazole. Antibody response against ES was measured by using ELISA. Solid line indicates cut-off OD value. (c) Scattergram of absorbance measured by ELISA to detect serum antibody against LMM antigen in children with neurocysticercosis before and after the treatment: from 33 clinically suspected and radiologically proven NCC children, serum samples were collected before and after 3–6 months and 12 months completion of the treatment with albendazole. Antibody response against LMM was measured by using ELISA. Solid line indicates cut-off OD value.

**Figure 2:**
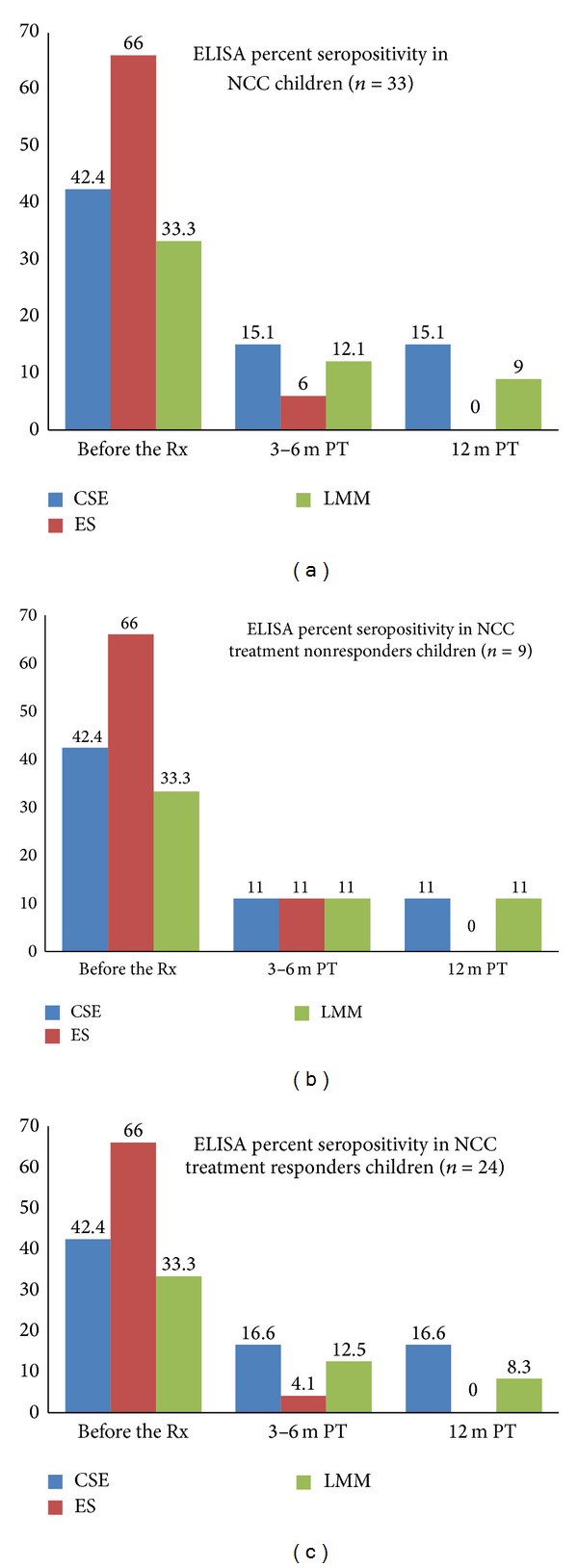
ELISA percent seropositivity in (a) NCC children before the treatment with albendazole, 3–6 months and 12 months after treatment. (b) NCC treatment nonresponders at 3–6 months and 12 months after treatment. (c) NCC treatment responders at 3–6 months and 12 months after treatment.

Antibody response to CSE, ES, and LMM antigenic fraction was positive in 14 (42.4%), 22 (66.6%), and 11 (33.3%) serum samples, respectively (Table 1 and Figures 1 and 2). Antibody response to ES antigen is significantly higher than CSE and LMM antigenic fractions. It is indicating that the sensitivity of ES antigen is significantly higher than CSE and LMM antigens to detect antibody in children with NCC.

#### 3.2.2. Serum after Treatment

After 3–6 months PT, antibody response to CSE, ES, and LMM antigenic fraction was positive in 5 (15.1%), 2 (6%), and 4 (12.1%) serum samples, and 12 months following the treatment, it was positive in 5 (15.1%), 0, and 3 (9%) patients, respectively (Table 1 and Figures 1 and 2). Statistical significance in serum antibody response to different antigenic fractions was compared in Table 1.

#### 3.2.3. Urine before Treatment

Antibody response to CSE, ES, and LMM antigenic fraction was positive in 20 (60.6%), 18 (54.5%), and 16 (48.4%) urine samples, respectively (Table 2). Statistical significance in urine antibody response to different antigenic fractions was compared in Table 2.

#### 3.2.4. Urine after Treatment

3–6 months PT, antibody response to CSE, ES, and LMM antigenic fraction was positive in 17 (51.5%), 12 (36.3%), and 16 (48.4%) samples, and 12 months following the treatment, it was positive in 16 (48.4%), 11 (33.3%), and 15 (45.4%) urine samples, respectively (Table 2). No significant difference in antibody positivity was found with the use of CSE and LMM antigens in the urine samples collected 3–6 months and 12 months PT in both responders and nonresponders, while significant difference was found with the use of ES antigen before the treatment, 3–6 months and 12 months PT (Table 2).

### 3.3. CT/MRI versus ELISA Positivity after Treatment

Out of 24 patients with resolving lesions on CT/MRI scans, 4 (16.6%) and 3 (12.5%) serum samples were positive at 3–6 months PT; 4 (16.6%) and 2 (8.3%) serum samples were persistently positive even 12 months PT with the use of CSE and LMM antigens, respectively. Out of 9 patients with persistent lesions in CT/MRI, one sample (11%) was positive at 3–6 months and 12 months PT, with the use of CSE/LMM antigens, while, with the use of ES antigen, only one sample was reactive after 3–6 months both in responder and in nonresponder groups, but none of the serum samples were reactive 12 months PT (Table 3).

## 4. Discussion

In the present study, the efficacy of three types of antigens (CSE, ES, and LMM) was assessed by ELISA to detect the posttreatment IgG antibody response in serum and urine sample collected from 33 NCC children following treatment with albendazole. ELISA is a simple and economical test, which compliments CT/MRI scans in the immunodiagnosis of NCC. Many studies have reported the PT (albendazole/praziquantel) radiological evolution of the lesions by indicating the decrease in number and size, total resolution or gliosis, and calcification [10, 32, 33]. In the present study, PT analysis of CT/MRI reports in 33 cases showed persistence of lesions in 27.2% (single cyst in 7 patients and multiple active cysts in 2 cases) of NCC cases at 3 months PT. This is in agreement with the previous report conducted in the same institute where disappearance of the lesion was reported in 75.7% of children 6 months PT with albendazole [32].

Very few reports indicate the evaluation of ELISA to detect humoral immune response in the assessment of therapeutic response after the treatment with the use of crude [12], purified, and heterologous antigens [13]. In the present study, with the use of crude antigen, out of 14 serum samples positive before the treatment, 5 (35.7%) samples were persistently seropositive even 3–6 months and 12 months PT. In our earlier study, evaluation of antibody responses to CSE antigen in PT serum samples was attempted in 10 NCC patients who reported 1, 3, and 6 months following albendazole treatment (15 mg/kg body weight for 15 days). In that study, out of 6 seropositive cases, 6 (100%), 6 (100%), and 3 (50%) serum samples were found to be persistently positive for IgG response to CSE antigen after 1, 3, and 6 months PT, respectively [12].

To the best of our knowledge, reports of use of ES antigen for the assessment of humoral antibody response in NCC patients after treatment are lacking. In the present study, with the use of ES antigen, out of 22 serum samples positive before the treatment, 2 (9%) (one responder and one nonresponder) serum samples were positive at 3–6 months PT, and none of the serum samples was reactive at 12 months PT. The reason for absence of antibody in serum 12 months following treatment may be due to the absence of the excretory secretory products from the inactive or calcified lesions, thereby indicating the seronegative response to the ES antigens in the serum samples [21]. The study suggests that the detection of antibody response in serum samples to ES antigens than CSE or purified antigenic fractions may be useful for the evaluation of therapeutic response in neurocysticercosis patients and positivity with the use of ES antigens may indicate the persistence of the active lesions in the brain.

In the present study, out of 11 serum samples positive before the treatment, with the use of 10–30 kDa antigenic fractions, 4 (36.3%) serum samples at 3–6 months PT and 3 (27.2%) samples at 12 months PT were persistently positive. In our earlier study, with the use of Sephadex G-200 purified antigenic fraction (PAF-II) of* C. cellulosae* in ELISA, the antibody response was found to be persistently positive in 4 (36.4%) out of 11 serum samples collected within 3–6 months of albendazole treatment [34]. In another study, in ELISA with the use of antigen B, out of 6 seropositive cases, 5 (83.3%), 3 (50%), and 1 (16.7%) serum samples had detectable IgG at 1, 3, and 6 months following treatment, respectively [12]. The results of the present study are in agreement with the recent study using the* Cysticercus fasciolaris* larval stage of* T. taeniaeformis *antigen. In that study, among the responders, 86.7% (IgG) and 79.5% (IgM) had converted to negative antibody titers at 6 months PT. Thirteen (81.2%) of 16 and 12 (80%) of 15 nonresponders continued to show high anti-cysticercus IgG and IgM titers, respectively. The study suggested that a negative ELISA result for both IgG and IgM antibodies denotes the cure of NCC [13]. In the present study, before the treatment, all of the serum samples positive with the CSE and LMM antigens were also positive with the use of ES antigens. In the case after treatment, the serum samples positive with the LMM antigens were also positive in ELISA with the CSE antigen.

In the present study, with the use of urine samples, there was no significant difference in the ELISA positivity with the use of crude/ES/10–30 kDa antigenic fractions for the detection of antibody at 3–6 months and 12 months PT. It indicates that the urine sample may not serve useful purpose for evaluation of therapeutic response in the neurocysticercosis patients. However, until now, no reports are available regarding evaluation of urine samples to assess the antibody response following treatment.

In the present study, although no significant correlation was found between the radiological findings at 3 months PT and antibody response at 3–6 months PT, there was significant correlation between the clinical outcome of the patient and the antibody response. Out of 9 patients with persistent lesions, none of them were seropositive at 12 months PT in ES ELISA. This supports the earlier reports that persistence of lesions solely in the brain does not necessarily constitute active infection [11]. In an earlier study from Mexico, direct relationship between the living stage of cysts and the presence of antibodies against E/S antigens has been reported [21].

In conclusion, the present study suggests that the presence of detectable antibody in serum against the excretory secretory antigens irrespective of their CT/MRI observation may indicate the active infection and also indicate the necessity to continue the treatment. The limitation of the present study was that CT/MRI of the NCC patients was available only 3 months after completion of the treatment. CT/MRI was not done 12 months following treatment and so correlation of the antibody response with the radioimaging reports 12 months after treatment was not possible.

## Figures and Tables

**Table 1:** Number of serum samples positive before the treatment, at 3–6 and 12 months following the treatment with the use of ∗CSE, ∗∗ES, and ∗∗∗LMM antigens.

Antigen	*n* = 33 (%)		
	Before Rx	3–6 months	12 months
∗CSE	14^a^ (42.4)	5^b^ (15.1)	5^c^ (15.1)
∗∗ES	22^d^ (66.6)	2^e^ (6)	0^f^
∗∗∗LMM	11^g^ (33.3)	4^h^ (12.1)	3^i^ (9)

*P* values a versus b; d versus e; g versus h; e versus f <0.05; b versus c; h versus i >0.05 (McNemar Test Exact).

∗CSE: crude soluble extract; ∗∗ES: excretory secretory; ∗∗∗LMM: lower molecular mass; *n* = number studied.

**Table 2:** Number of urine samples positive before the treatment and at 3–6 and 12 months after treatment with the use of CSE, ES, and LMM antigens in ELISA (%).

Antigen	NCC = 33 (%)		
	Before Rx	After 3–6 months	After 12 months
CSE	20^a^ (60.6)	17^b^ (51.5)	16^c^ (48.4)
ES	18^d^ (54.5)	12^e^ (36.3)	11^f^(33.3)
LMM	16^g^ (48.4)	16^h^ (48.4)	15^i^ (45.4)

*P* values a versus b and c; d versus e and f; g versus h and i = >0.05; d versus f = <0.05

**Table 3:** Posttreatment radiological reports versus antibody response before the treatment, 3–6 months and 12 months following the treatment.

Radiological findings *n* = 33	Serum antibody response					
	Number positive (%)					
	CSE		ES		LMM	
	3–6 mth	12 mth	3–6 mth	12 mth	3–6 mth	12 mth
Resolved lesion (*n* = 24)	4 (16.6)	4 (16.6)	1 (4.1)	N.D	3 (12.5)	2 (8.3)
Persistent lesion (*n* = 9)	1 (11)	1 (11)	1 (11)	N.D	1 (11)	1 (11)

N.D: not in detectable levels; mth: month; *n*: number studied.
